# Erlotinib overcomes paclitaxel-resistant cancer stem cells by blocking the EGFR-CREB/GRβ-IL-6 axis in MUC1-positive cervical cancer

**DOI:** 10.1038/s41389-019-0179-2

**Published:** 2019-11-26

**Authors:** Yaping Lv, Wei Cang, Quanfu Li, Xiaodong Liao, Mengna Zhan, Huayun Deng, Shengze Li, Wei Jin, Zhi Pang, Xingdi Qiu, Kewen Zhao, Guoqiang Chen, Lihua Qiu, Lei Huang

**Affiliations:** 1 Department of Histoembryology, Genetics and Developmental Biology, Key Laboratory of Cell Differentiation and Apoptosis of Chinese Ministry of Education, Shanghai Key Laboratory of Reproductive Medicine, Shanghai Jiao Tong University School of Medicine, Shanghai, P. R. China; 2 Department of Gynecology and Obstetrics, Shanghai Key Laboratory of Gynecologic Oncology, Renji Hospital, School of Medicine, Shanghai Jiao Tong University, Shanghai, P. R. China; 3 Department of Pathology, Zhongshan Hospital, Fudan University, Shanghai, P. R. China; 4 Key Laboratory of Cell Differentiation and Apoptosis of The Chinese Ministry of Education, Department of Pathophysiology, Shanghai Jiao Tong University School of Medicine, Shanghai, China

**Keywords:** Cancer stem cells, Cancer therapeutic resistance, Growth factor signalling, Cancer microenvironment, Targeted therapies

## Abstract

Cancer stem cells (CSCs) are often enriched after chemotherapy and contribute to tumor relapse. While epidermal growth factor receptor (EGFR) tyrosine kinase inhibitors (TKIs) are widely used for the treatment of diverse types of cancer, whether EGFR-TKIs are effective against chemoresistant CSCs in cervical cancer is largely unknown. Here, we reveal that EGFR correlates with reduced disease-free survival in cervical cancer patients with chemotherapy. Erlotinib, an EGFR-TKI, effectively impedes CSCs enrichment in paclitaxel-resistant cells through inhibiting IL-6. In this context, MUC1 induces CSCs enrichment in paclitaxel-resistant cells via activation of EGFR, which directly enhances IL-6 transcription through cAMP response element-binding protein (CREB) and glucocorticoid receptor β (GRβ). Treatment with erlotinib sensitizes CSCs to paclitaxel therapy both in vitro and in vivo. More importantly, positive correlations between the expressions of MUC1, EGFR, and IL-6 were found in 20 cervical cancer patients after chemotherapy. Mining TCGA data sets also uncovered the expressions of MUC1-EGFR-IL-6 correlates with poor disease-free survival in chemo-treated cervical cancer patients. Collectively, our work has demonstrated that the MUC1-EGFR-CREB/GRβ axis stimulates IL-6 expression to induce CSCs enrichment and importantly, this effect can be abrogated by erlotinib, uncovering a novel strategy to treat paclitaxel-resistant cervical cancer.

## Introduction

Cancer stem cells (CSCs), featured with self-renewal and differentiation abilities are responsible for tumor initiation, metastasis and recurrence upon chemotherapy^1^. CSCs are often enriched after chemotherapy and induce tumor recurrence, which poses a significant clinical challenge^2,3^. Similarly, cervical CSCs resistant to chemotherapy^4^ make this subpopulation as a critical target for cervical cancer treatment. Various mechanisms of the enrichment of CSCs have been reported, including (1) high expression of ABC transporters that pump drugs out from tumor cells^5^, (2) enhanced DNA repair capacity^6^, and (3) resistance to apoptosis^7^. In addition, growing evidences indicate a crucial role of various cytokines/chemokines in mediating chemoresistance of CSCs, such as interleukin-6 (IL-6) and interleukin-8 (IL-8)^8,9^.

Cervical cancer ranks fourth among the most common malignant tumors in women worldwide^10^. With the progress of prevention and treatment, the prognosis of cervical cancer patients has improved. However, it is not fully curative for advanced cervical cancer due to recurrence, metastasis, or lack of an optimal treatment option^11,12^. Neoadjuvant chemotherapy (NACT) has been used for treatment of cervical cancer, mainly for patients with advanced cervical cancer, which is usually a combination of platinum and paclitaxel^13^. However, it has been reported that NACT followed by surgery has no therapeutic advantage compared with standard treatment^14,15^. Therefore, the treatment of NACT is not included in national comprehensive cancer network (NCCN) guidelines for cervical cancer treatment. Hence, the development of targeted and effective therapies is imperative and of enormous clinical significance. Paclitaxel is one of the first-line chemotherapeutic agents for recurrent or metastatic cervical cancer^16^. CSCs resistance to paclitaxel has been reported. For instance, ovarian CSCs were shown to be enriched upon paclitaxel treatment^17^. CSCs of breast cancer were also expanded by paclitaxel in a HIF-1-dependent manner^18^. Another study reported that the percentage of human oral squamous CSCs was increased by paclitaxel treatment in a concentration-dependent manner^19^. Since tumors that recurred after chemotherapy tend to be more aggressive, there is an urgent need to understand the mechanism underlying paclitaxel-induced CSCs enrichment and to develop an efficient strategy to inhibit paclitaxel-resistant CSCs.

Epidermal growth factor receptor (EGFR) is a member of transmembrane tyrosine kinase receptor family and widely expressed in human cancers^20^. EGFR is autophosphorylated after binding to the corresponding ligand and activates downstream signaling pathways critical to myriad processes, such as cell proliferation, angiogenesis, and CSCs enrichment^21,22^. EGFR is also found in the nucleus of carcinoma cells where it functions as a coactivator to regulate the transcription of several genes^23^. Epidermal growth factor receptor tyrosine kinase inhibitors (EGFR-TKIs) have been reported to be effective in the treatment of breast^24^, head and neck^25^, and colorectal cancer^26^, especially for patients with EGFR mutation. EGFR-TKIs block autophosphorylation of EGFR by binding to the intracellular tyrosine kinase domain^27^. Erlotinib is a selective EGFR-TKI, which is routinely used as standard therapy for advanced EGFR mutation-positive non-small cell lung cancer^28^ or in combination with gemcitabine for treatment of pancreatic cancer^29^. However, little is known about the effect of erlotinib on paclitaxel-resistant cervical CSCs.

We recently reported that erlotinib could enhance the sensitivity of MUC1-expressing cancer cells to paclitaxel^30^. We extended the study by investigating whether paclitaxel-induced CSC enrichment could be affected by erlotinib. We demonstrate that erlotinib can effectively inhibit paclitaxel-resistant CSCs and reveal the mechanism of action.

## Results

### EGFR correlates with reduced disease-free survival in cervical cancer patients with chemotherapy

**Fig. 1:**
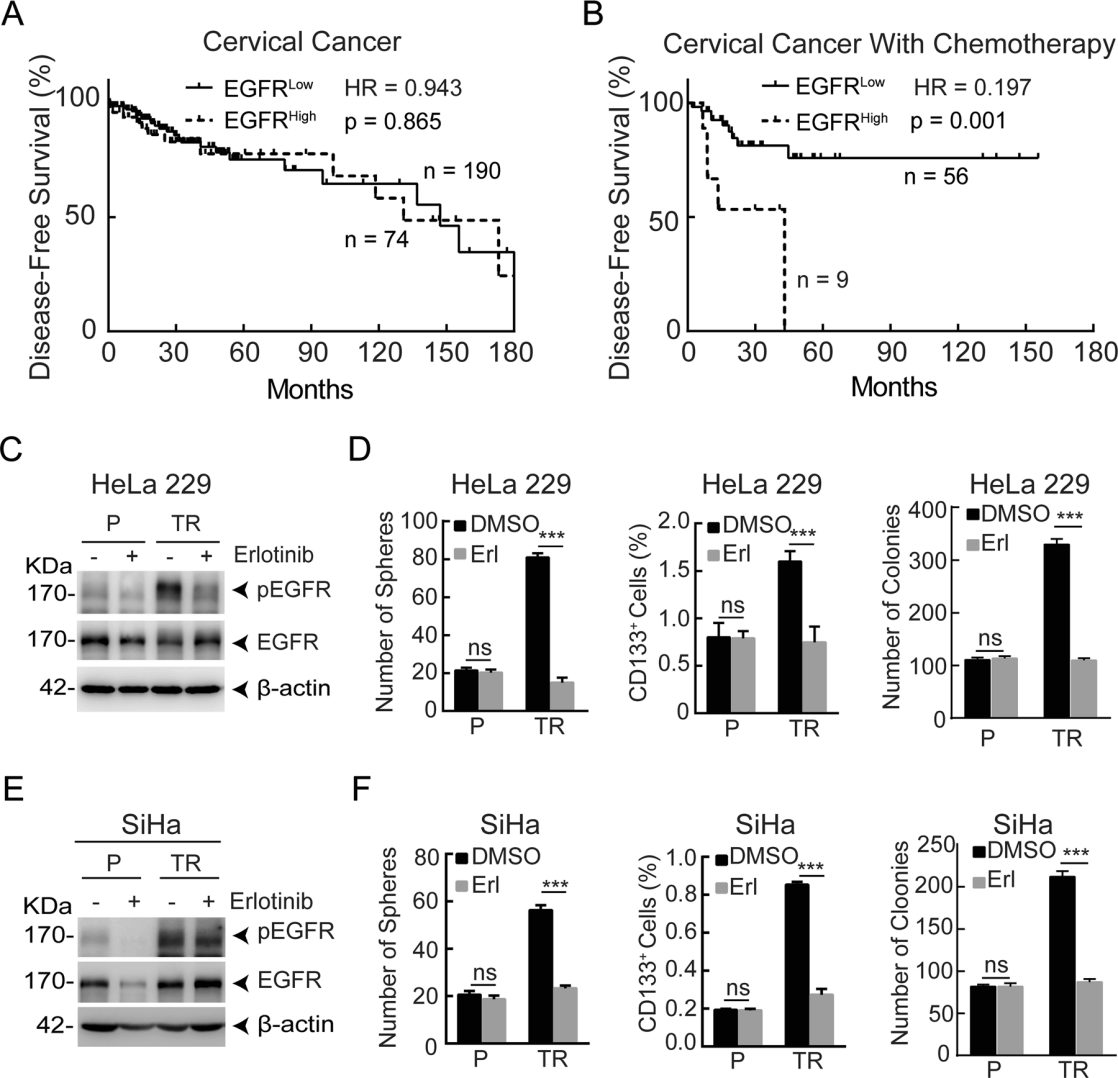
EGFR correlates with reduced disease-free survival in cervical cancer patients with chemotherapy and EGFR suppression reduces CSCs enrichment in paclitaxel-resistant cervical cancer cells. **a**, **b** Analysis of the relationship between the high expression of EGFR (the mRNA expression of EGFR exceeds the average value of the test samples) and disease-free survival in cervical cancer patients (TCGA, *n* = 264) (**a**) and cervical cancer patients with chemotherapy (TCGA, *n* = 65) (**b**). **c** Western blot analysis was performed to detect the protein levels of phosphorylation of EGFR (pEGFR) and EGFR in HeLa229P and HeLa229/TR cells after treated with 0 μM or 5 μM erlotinib for 48 h. **d** Sphere-formation assay, flow cytometry assay of CD133, and colony-forming assay were performed in HeLa229P and HeLa229/TR cells with or without 5 μM erlotinib treatment for 48 h. **e** The protein levels of pEGFR and EGFR in SiHaP and SiHa/TR cells with or without 5 μM erlotinib treatment for 48 h were measured by western blot. **f** The spheres number, the percentage of CD133^+^ cells, and the colonies number in SiHaP and SiHa/TR cells with or without 5 μM erlotinib treatment for 48 h were detected. Data represent mean ± SD from three independent experiments (*n* = 3). Differences between linked groups were evaluated by two-tailed Student’s *t* test. ***P < 0.001; ns not significant, P parental, TR paclitaxel-resistant, Erl erlotinib.

To explore a potential relationship between the expression of EGFR and the clinical outcome of cervical cancer patients, we mined TCGA data sets, and found that the expression of EGFR had little if any correlation with the disease-free survival of cervical cancer patients (Fig. 1a). However, the elevated expression of EGFR was significantly associated with poor disease-free survival in chemo-treated cervical cancer patients (Fig. 1b). These results suggest a potential role of EGFR in chemotherapy resistance.

### Erlotinib suppresses CSCs enrichment in paclitaxel-resistant cervical cancer cells

CSCs play an essential role in tumor recurrence after chemotherapy. Numerous studies reveal that CD133^+^ cells possess the ability to form cell spheres, express high levels of stem cell-related genes, and have an enhanced tumor-formation potential^31–33^. Given that the TCGA analyses showed that EGFR status was associated with disease-free survival in cervical cancer patients with chemotherapy, we sought to test whether erlotinib could be a useful agent to target CSCs. Consistent with our previous report^30^, the phosphorylation of EGFR (pEGFR, a surrogate maker of EGFR activity) was considerably increased in HeLa229 paclitaxel-resistant cells (HeLa229/TR) compared with parental cells (HeLa229P). In accordance, the levels of pEGFR were substantially reduced upon treatment of the cells with erlotinib (Fig. 1c). Since CSCs can generate multicellular spheroids in serum-free medium^34^, we performed the sphere-formation assay to detect CSCs population in paclitaxel-resistant cells. Indeed, relative to HeLa229P cells, there was a huge increase in spheres number in HeLa229/TR cells, and this increase was completely diminished upon erlotinib treatment (Fig. 1d left; Supplementary Fig. S1A). To corroborate the sphere data, we performed flow cytometry (FCM) assay and colony-forming assay. The results showed that erlotinib treatment significantly reduced the percentage of CD133^+^ cells and the number of colonies in HeLa229/TR cells, and had little if any effect on that of HeLa229P cells (Fig. 1d middle, 1d right; Supplementary Fig. S1B). A parallel experiment was performed in SiHa and its paclitaxel-resistant cell line SiHa/TR, which showed a similar association between EGFR activity and CSCs enrichment (Fig. 1e, f; Supplementary Fig. S1C, D). To further substantiate the role of erlotinib in CSCs enrichment, we knocked down the expression of EGFR using shRNA. Diminished expression of EGFR (Supplementary Fig. S1E) was associated with significant reduction of the number of spheres, the percentage of CD133^+^ cells, and the number of colonies in paclitaxel-resistant cell (Supplementary Fig. S1F). These results together demonstrate that erlotinib effectively suppresses CSCs enrichment in paclitaxel-resistant cervical cancer cells.

### Erlotinib prevents cervical CSCs enrichment through inhibiting IL-6

**Fig. 2:**
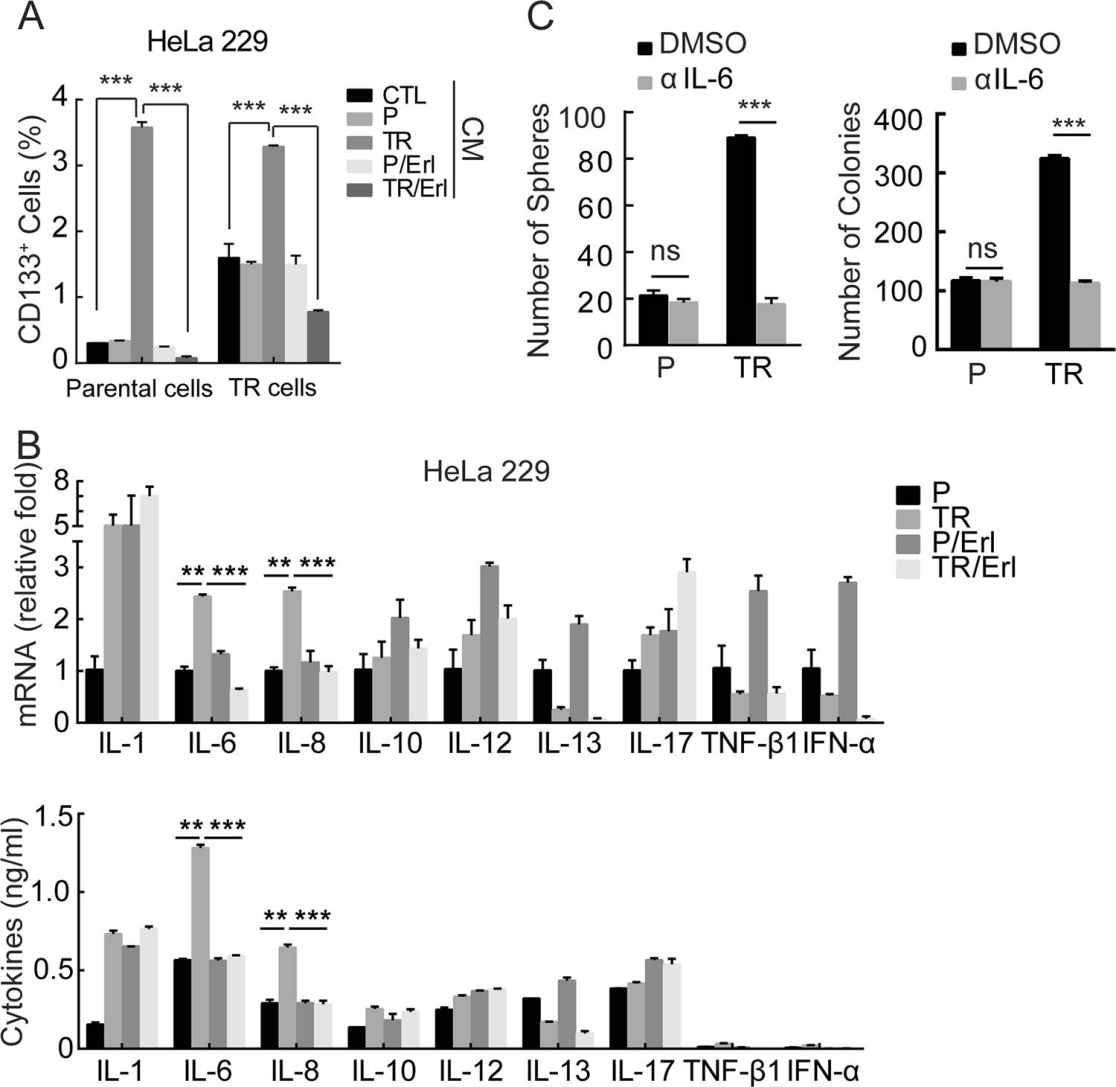
Erlotinib prevents cervical CSCs enrichment through inhibiting IL-6. **a** Collection of conditional medium form HeLa229P or HeLa229/TR cells with or without 5 μM erlotinib treatment for 48 h to culture HeLa229P or HeLa229/TR cells, then flow cytometry assay was applied to detect the percentage of CD133^+^ cells. **b** RT-qPCR (upper) and ELISA (lower) analysis of indicated cytokines in HeLa229P and HeLa229/TR cells after treated with 0 μM or 5 μM erlotinib for 48 h. **c** Sphere-formation assay and colony-forming assay were performed in HeLa229P and HeLa229/TR cells treated with DMSO or IL-6 neutralizing antibody (10 μg/ml) for 48 h (scale bar = 100 µm). Data represent mean ± SD from three independent experiments (*n* = 3). Differences between linked groups were evaluated by two-tailed Student's *t* test. **P < 0.01; ***P < 0.001; ns not significant, P parental, TR paclitaxel-resistant, CM conditional medium, Erl erlotinib, αIL-6 IL-6 neutralizing antibody.

We previously found that erlotinib alleviated chemoresistance by suppressing the expression of ABCB1^30^. However, silencing ABCB1 had little if any effect on the enrichment of CSCs in paclitaxel-resistant cells (data not shown). Recent studies have demonstrated a critical role of various cytokines in regulation of CSCs^35,36^. We therefore asked if erlotinib might impede CSCs enrichment through inhibiting cytokines secretion. To this end, the conditional medium from HeLa229P or HeLa229/TR cells with or without erlotinib treatment was applied to the culture of HeLa229P and HeLa229/TR cells (Supplementary Fig. S2A). FCM analysis showed that HeLa229/TR conditional medium significantly enriched the CD133^+^ cells population in both HeLa229P and HeLa229/TR cells. In addition, HeLa229 TR/Erl but not HeLa229P/Erl conditional medium significantly reduced the CD133^+^ cells population in both HeLa229P and HeLa229/TR cells (Fig. 2a; Supplementary Fig. S2B). This result suggested that erlotinib might hamper CSCs enrichment through inhibiting cytokines production. To search for cytokines involved in this effect, we screened nine cytokines that were known to contribute to CSCs^37,38^ in HeLa229P and HeLa229/TR cells. Among the cytokines tested, we found that both mRNA (Fig. 2b upper) and protein (Fig. 2b lower) levels of IL-6 and IL-8 were significantly upregulated in HeLa229/TR cells compared with HeLa229P cells. Conversely, both IL-6 and IL-8 were dramatically reduced by erlotinib treatment (Fig. 2b). To test whether chemotherapy induced CSCs enrichment was mediated by IL-6 or IL-8, we treated cells with neutralizing antibodies against each cytokine respectively. Interestingly, IL-6 (Fig. 2c; Supplementary Fig. S2C) but not IL-8 (Supplementary Fig. S2D) neutralizing antibody abrogated the enrichment of CSCs indicating by spheres and colonies in paclitaxel-resistant cells, but not in parental cells. Similar results were obtained in SiHa parental and TR cell lines (Supplementary Fig. S2E, F). These data collectively reveal that erlotinib blocks cervical CSCs enrichment through inhibiting IL-6.

### MUC1 activates EGFR to promote IL-6 expression and CSCs enrichment

Our previous study showed that EGFR was involved in MUC1-induced chemotherapy resistance^30^, which led us to hypothesize that EGFR activity might play a role in MUC1-mediated CSCs enrichment via regulation of IL-6 expression. To test this, we knocked out *MUC1* gene in HeLa229/TR (Supplementary Fig. S3A) and SiHa/TR (Supplementary Fig. S3F) cells through CRISPR/Cas9. Remarkably, MUC1 deficiency resulted in a substantial reduction in not only mRNA expression of IL-6 (Supplementary Fig. S3B left) and production of IL-6 (Supplementary Fig. S3B right) but also spheres number (Supplementary Fig. S3C) and colonies number (Supplementary Fig. S3D) in HeLa229/TR cells. These data suggest that paclitaxel-induced CSCs was mediated by MUC1. We next examined the effect of *MUC1* knockout on activation of EGFR and found that the pEGFR was significantly decreased in MUC1-deficient HeLa229/TR cells (Supplementary Fig. S3A). In accordance, the levels of pEGFR, IL-6, the number of spheres, and the number of colonies were significantly reduced upon treatment of erlotinib in HeLa229 TR/CTL cells, but not in HeLa229 TR/CRISPR cells (Supplementary Fig. S3A–D). Moreover, IL-6-neutralizing antibody effectively abrogated the paclitaxel-induced sphere formation in HeLa229 TR/CTL cells, but not in HeLa229 TR/CRISPR cells (Supplementary Fig. S3E). A similar experimental strategy was employed with SiHa/TR cells, which showed an analogous association among MUC1 expression, EGFR activation, IL-6 expression, and CSCs enrichment (Supplementary Fig. S3F–I). To further verify the role of the MUC1-EGFR-IL-6 axis in paclitaxel-resistance, we knocked down *MUC1* in HeLa229 parental cells and found that chemotherapy-induced pEGFR expression was abolished (Supplementary Fig. S4A). In addition, paclitaxel failed to stimulate IL-6 expression in erlotinib-treated cells (Supplementary Fig. S4B). Furthermore, we applied the conditional medium from HeLa229/shCTL or HeLa229/shMUC1-B cells with or without paclitaxel treatment to the cultures of HeLa229/shCTL and HeLa229/shMUC1-B cells (Supplementary Fig. S4C upper). Paclitaxel-treated HeLa229/shCTL conditional medium significantly expanded the CD133^+^ cells population in HeLa229/shCTL cells, but not in HeLa229/shMUC1-B cells (Supplementary Fig. S4C lower). These data suggested that MUC1 promotes CSCs enrichment through stimulating IL-6-mediated autocrine effect. Accordingly, paclitaxel failed to induce spheres and colonies formation in the presence of erlotinib or IL-6 neutralizing antibody in HeLa229/shCTL cells (Supplementary Fig. S4D–E). Altogether, these results demonstrate that MUC1 activates EGFR to promote IL-6 expression and CSCs enrichment.

### EGFR induces IL-6 transcription through CREB and GRβ binding sites

**Fig. 3:**
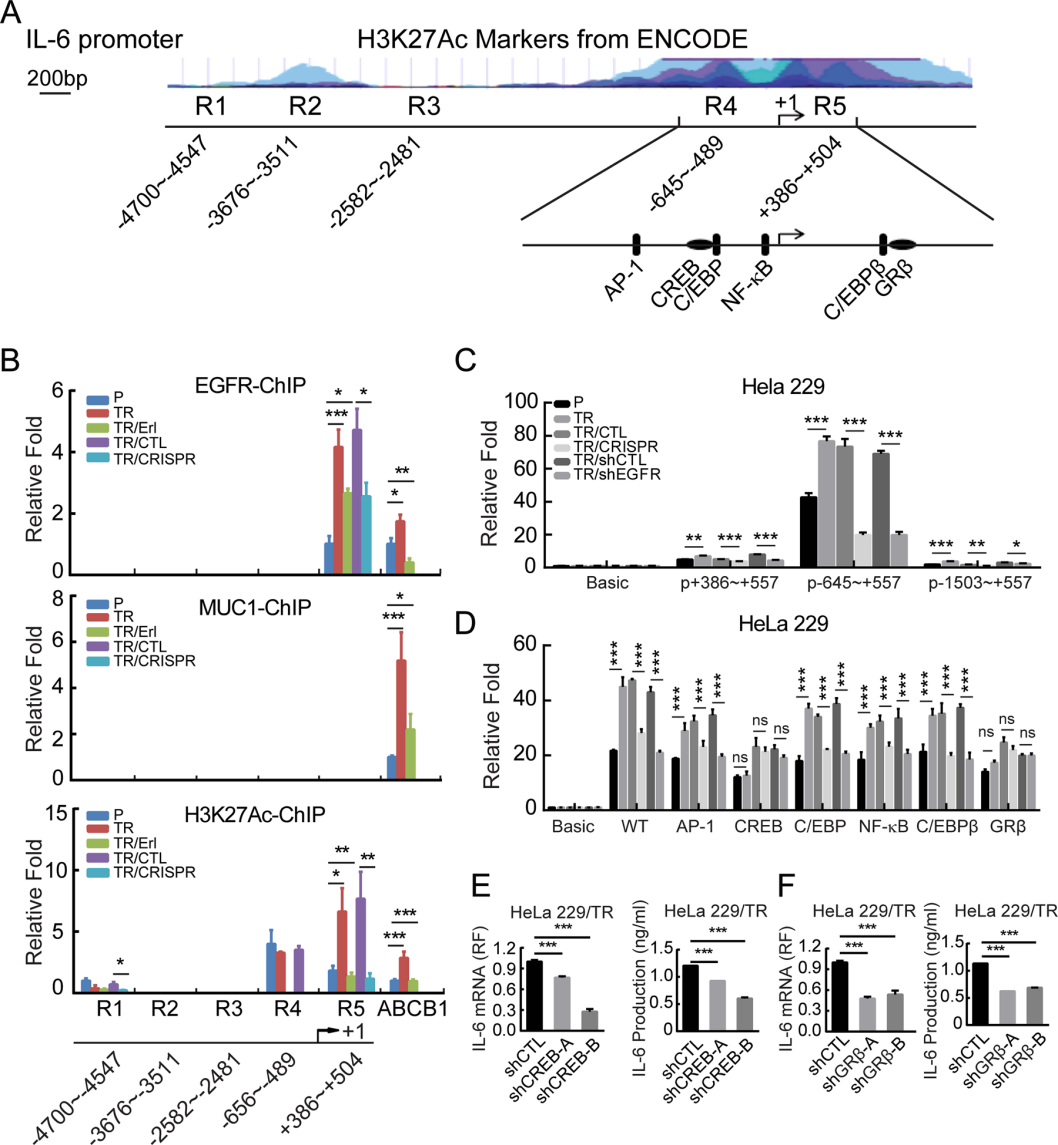
EGFR induces IL-6 transcription through CREB and GRβ binding sites. **a** Schematic illustration of the H3K27Ac (Histone H3acetyl K27) enrichment and PCR fragments of the human *IL-6* gene promoter using the UCSC genome browser. **b** Chromatin immunoprecipitation (ChIP) assays were performed in HeLa229P, HeLa229/TR, HeLa229 TR/Erl, HeLa229 TR/CTL, and HeLa229 TR/CRISPR cells using antibodies against MUC1, EGFR, and H3K27Ac, respectively, to identify the occupancy on IL-6 gene promoter. The ABCB1 promoter was used as a positive control for ChIP. **c** HeLa229P, HeLa229/TR, HeLa229 TR/CTL, HeLa229 TR/CRISPR, HeLa229 TR/shCTL, and HeLa229 TR/shEGFR cells were transfected with pGL3-IL-6 promoters or pGL3-basic plasmids (500 ng) and pGL3-renilla plasmid (10 ng), luciferase activity was detected. **d** HeLa229P, HeLa229/TR, HeLa229 TR/CTL, HeLa229 TR/CRISPR, HeLa229 TR/shCTL, and HeLa229 TR/shEGFR cells were transfected with pGL3-IL-6-mutant-promoters or pGL3-basic plasmids (500 ng) and pGL3-renilla plasmid (10 ng) and subjected to luciferase activity assay. The bars in **d** is the same as that in **c** chart. **e** The mRNA levels (left) and production (right) of IL-6 in HeLa229 TR/shCTL and HeLa229 TR/shCREBs were measured. **f** The mRNA levels (left) and production (right) of IL-6 in HeLa229 TR/shCTL and HeLa229 TR/shGRβs cells were detected. Data represent mean ± SD from three independent experiments (*n* = 3). Differences between linked groups were evaluated by two-tailed Student's *t* test. *P < 0.05; **P < 0.01; ***P < 0.001; ns not significant, P parental, TR paclitaxel-resistant, TR/Erl paclitaxel-resistant/erlotinib, p+386~+557: pIL-6-promoter-+386~+557, p-645~+557: pIL-6-promoter-645~+557, p-1503~+557: pIL-6-promoter-1503~+557, WT: pGL3-IL-6-Luc-wt, AP-1: pGL3-IL-6-AP-1-mutant, NF-κB: pGL3-IL-6-NF-κB-mutant, C/EBP: pGL3-IL-6-C/EBP-mutant, CREB: pGL3-IL-6-CREB-mutant, C/EBPβ: pGL3-IL-6-C/EBPβ-mutant, GRβ: pGL3-IL-6-GRβ-mutant.

To determine how MUC1-EGFR is involved in IL-6 regulation, we conducted immunofluorescence staining in HeLa229P and HeLa229/TR cells that were treated with or without erlotinib (Supplementary Fig. S5A), as well as HeLa229/shMUC1-B cells that re-expressed MUC1 and were treated with or without paclitaxel respectively (Supplementary Fig. S5B). Consistent with our previous report, we found that paclitaxel treatment increased both MUC1 and EGFR in the nucleus, and this effect was blocked by treatment with erlotinib. In light of many studies showing that MUC1 and EGFR act as transcription coactivators, we investigated whether MUC1 and EGFR in the nucleus might participate in the transcriptional regulation of IL-6. Chromatin immunoprecipitation (ChIP) showed that paclitaxel induced the binding of EGFR to IL-6 promoter around the region of +386 to +504 (Fig. 3a). This effect of paclitaxel was completely abolished by EGFR inhibitor or MUC1 depletion (Fig. 3b upper). Interestingly, we found that MUC1 bound to the ABCB1 promoter, as expected, but not IL-6 promoter (Fig. 3b middle). The H3K27Ac acts as a transcriptional activation control (Fig. 3b lower). The results suggested that the effect of MUC1 on IL-6 was mediated by EGFR, which directly bound to IL-6 promoter. We carried out luciferase assay to further assess the effect of EGFR. IL-6-promoter-driven luciferase activity was drastically elevated in HeLa229/TR cells, within the region of −645~+557 in particular (Fig. 3c). Depletion of either MUC1 or EGFR markedly reduced the luciferase activity in HeLa229/TR cells (Fig. 3c), consistent with the notion that EGFR is downstream of MUC1. To further define the binding site of EGFR on IL-6 promoter, we mutated the four binding sites for AP-1, NF-κB^39^, C/EBP^40^, CREB^41^, and additional two predicted binding sites for C/EBPβ and GRβ within −645~+557 region (Fig. 3a). The luciferase activities were considerably reduced by mutation of binding sites for CREB and GRβ in HeLa229/TR cells, but had little additional change with MUC1 depletion and EGFR inhibition (Fig. 3d). To corroborate the role of CREB and GRβ in IL-6 transcriptional regulation, we silenced the expression of CREB and GRβ in HeLa229/TR cells (Supplementary Fig. S5C, D). Notably, depletion of CREB (Fig. 3e) or GRβ (Fig. 3f) significantly reduced the expression of IL-6 in paclitaxel-resistant cell. Altogether, these results indicated that EGFR stimulates IL-6 transcription by directly binding to CREB and GRβ sites.

### Coadministration of EGFR inhibitor with paclitaxel prevents MUC1-expressing tumor relapse

We next sought to investigate whether the MUC1-EGFR-IL-6 axis could be the potential therapeutic target to overcome paclitaxel-resistance. Xenograft mouse models were created by subcutaneously implanting HeLa229/shCTL or HeLa229/shMUC1 cells into dorsal flanks of nude mice. We found that tumor growth of HeLa229/shCTL implant were initially inhibited by paclitaxel treatment, but rapidly regrew after paclitaxel treatment was suspended (Supplementary Fig. S6A, B). In contrast, HeLa229/shMUC1-derived tumors were sensitive to paclitaxel treatment, and did not resume growth even after paclitaxel treatment was ceased (Supplementary Fig. S6A, B). Consistent with our in vitro data, the levels of MUC1 and EGFR, especially those in the nucleus (Supplementary Fig. S6C) as well as IL-6 (Supplementary Fig. S6D left and middle) were induced in paclitaxel-treated HeLa229/shCTL tumors, but not in HeLa229/shMUC1 tumors. FCM analysis of freshly isolated single-cell suspension from tumors revealed that paclitaxel dramatically induced an increase of CD133^+^ cells in HeLa229/shCTL tumors (Supplementary Fig. S6D right).

**Fig. 4:**
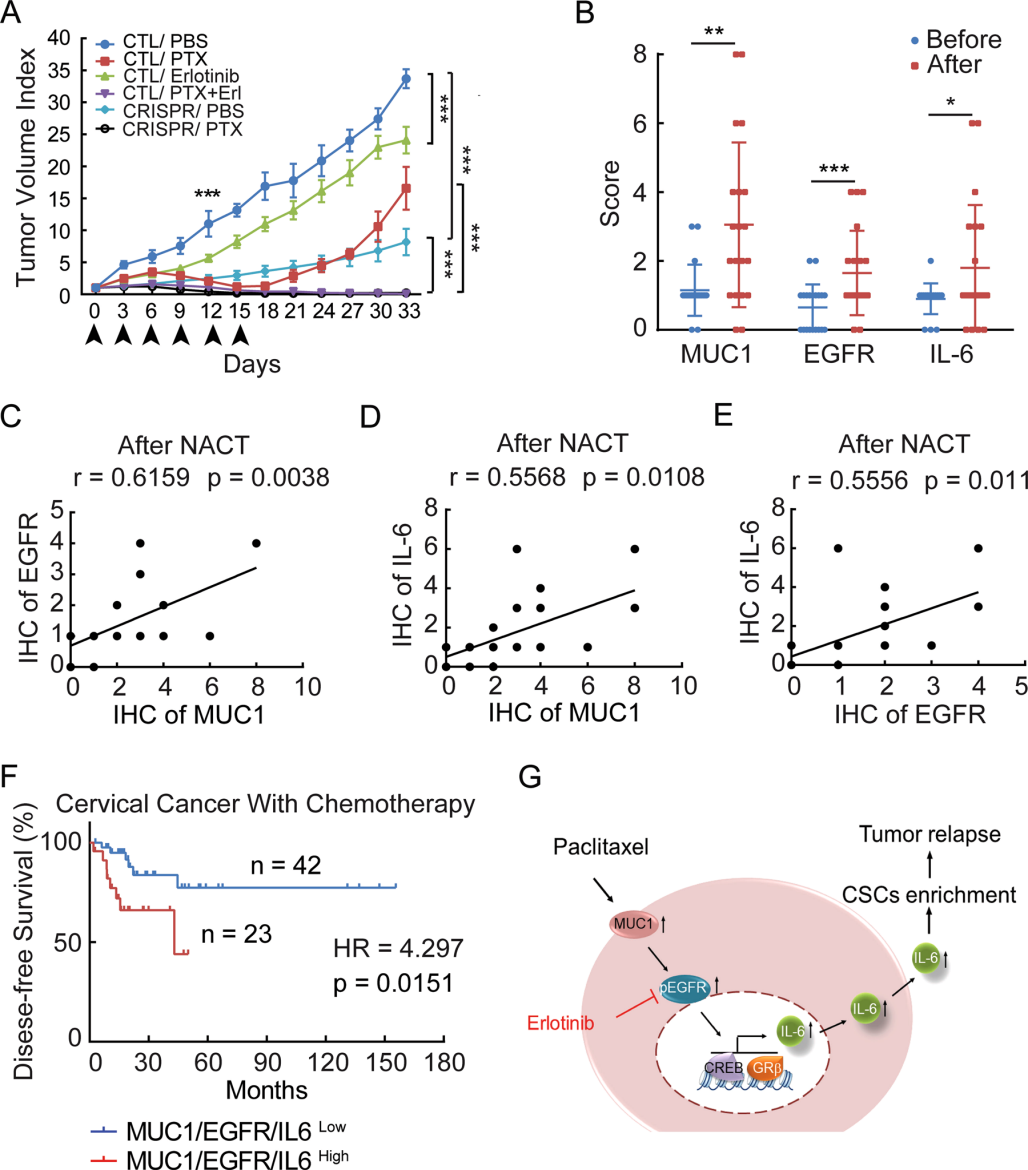
Activation of MUC1-EGFR-IL-6 signaling triggers xenograft tumor relapse and positively correlates with poor outcome of cervical cancer patients with chemotherapy. **a** HeLa229 TR/CTL or HeLa229 TR/CRISPR cells were subcutaneously injected to nude mice. When the tumors reached 4 mm × 4 mm, the mice were randomized to groups (*n* = 6 per group), and injected drugs as indicated intraperitoneally every 3 days for 15 days and the tumor volume was calculated. **b** IHC score of MUC1, EGFR, and IL-6 expression of pre- and post-NACT cervical cancer tissues are shown (*n* = 20). **c**–**e** Analysis of correlation between the IHC score of MUC1 and EGFR (**c**), MUC1 and IL-6 (**d**), EGFR and IL-6 (**e**) in 20 paired cervical cancer samples with NACT. **f** Disease-free survival study of MUC1-EGFR-IL-6 (patients with high expression of at least one of the three genes) was analyzed in cervical cancer patients with chemotherapy information from TCGA data sets. (*n* = 65). **g** Schematic overview of the MUC1-EGFR-CREB/GRβ axis stimulates IL-6 expression to induce CSCs enrichment and tumor relapse, this effect can be abrogated by erlotinib in paclitaxel-resistant cervical cancer. Differences between linked groups were evaluated by two-tailed Student's *t* test. *P < 0.05; **P < 0.01; ***P < 0.001; PTX paclitaxel, Erl erlotinib, NACT neoadjuvant chemotherapy.

We went on testing the effect of targeting EGFR or MUC1 on paclitaxel-resistant tumors. HeLa229 TR/CTL, or HeLa229 TR/CRISPR cells were implanted subcutaneously into nude mice. Although early treatment with paclitaxel alone significantly reduced HeLa229 TR/CTL tumors, tumor growth quickly recovered after discontinuation of paclitaxel treatment. In contrast, the combination of paclitaxel and erlotinib not only eliminated the growth of primary tumors but also prevented tumor recurrence. In mice implanted with HeLa229 TR/CRISPR cells, paclitaxel monotherapy significantly inhibited tumor growth and recurrence, suggesting that silencing of MUC1 sufficiently increased sensitivity of tumors to paclitaxel (Fig. 4a; Supplementary Fig. S6E). What is more, we found that combination therapy significantly inhibits tumor weight compared with either drug alone, but has little if any effect on body weight (Supplementary Fig. S6F). Taken together, these data indicate that the combination of targeting either EGFR or MUC1 and paclitaxel blocks paclitaxel-resistant CSCs, and thereby prevents recurrence of MUC1-positive cancer.

### Activation of MUC1-EGFR-IL-6 signaling correlates with poor disease-free survival of cervical cancer patients with chemotherapy

To examine the clinical relevance of the MUC1-EGFR-IL-6 axis in cancer patients, we collected 20 paired pre- and post-NACT tumor specimens from cervical cancer patients (Supplementary Table S6) and performed immunohistochemistry to detect the expression of MUC1, EGFR, and IL-6. Notably, the expression of MUC1 and EGFR, especially those in the nucleus, as well as IL-6 were upregulated in post-NACT specimens (Fig. 4b; Supplementary Fig. S7A, 7B). More importantly, the positive association of MUC1, EGFR, and IL-6 was more apparent after NACT (Fig. 4c–e) rather than in pre-NACT tissues (Supplementary Fig. S7C). We further analyzed 65 cervical cancer patients with chemotherapy information from TCGA data sets. The combined high expression of MUC1 and/or EGFR and/or IL-6 (patients with high expression of at least one of the three genes) (*n* = 23) was significantly associated with poor disease-free survival (Fig. 4f). Collectively, the data from cancer patients are in line with the notion that activation of MUC1-EGFR-IL-6 signaling correlated with poor outcome of cervical cancer patients with chemotherapy.

## Discussion

CSCs are the root cause of conventional chemotherapy failure, and currently is lack of effective treatment targeting chemoresistant CSCs. EGFR-TKIs have proven efficacy in multiple types of tumors^25,42^. However, little is known about the effect of EGFR-TKIs on paclitaxel-resistant cervical CSCs. Mining TCGA database uncovered that high expression of EGFR was significantly associated with poor disease-free survival in cervical cancer patients with chemotherapy (Fig. 1a, b). Supportive of this association, we showed that paclitaxel-resistant CSCs were highly enriched in both EGFR-mutated paclitaxel-resistant HeLa229 and SiHa cervical cancer cells^43^. Importantly, using complementary pharmacological and genetic approaches, we demonstrated that EGFR inhibition could reduce paclitaxel-resistant cervical CSCs (Fig. 1c–f). These data indicate a vital role of EGFR in paclitaxel-resistant cervical CSCs, carrying potential therapeutic implication.

Our previous study demonstrated that paclitaxel treatment promoted the activation of EGFR to upregulate ABCB1, subsequently led to chemotherapy resistance^30^. However, we further found that ABCB1 was not responsible for the enrichment of CSCs in paclitaxel-resistant cells, which led us to uncover an alternative mechanism underlying EGFR induces CSCs enrichment. We demonstrated that CSCs were induced by IL-6, consistent with published results indicating IL-6 as the master regulator of CSCs-inducing cytokines^44^. The critical contribution of IL-6 to cervical CSCs was evidenced by using IL-6 neutralizing antibody that blocked EGFR-induced cervical CSCs enrichment (Fig. 2c).

MUC1 is a high-molecular-weight transmembrane glycoprotein that is abnormally overexpressed in multiple cancers. Its abnormal expression is associated with tumor malignancy and poor prognosis, including cervical cancer^45^. MUC1 was shown to play an oncogenic role in regulation of self-renewal of CSCs in a variety of tumor cells^46,47^. Our previous study demonstrated that paclitaxel treatment induced MUC1 expression transcriptionally and post-translationally, which promoted the increase of nuclear EGFR, subsequently led to chemotherapy resistance^30^. These observations led us to ask whether EGFR could mediate CSCs enrichment in the context of MUC1 expression. Indeed, by using a loss-of-function approach, we confirmed that activation of the EGFR-IL-6 pathway and enrichment of paclitaxel-resistant CSCs depended on MUC1 expression in cervical cancer (Supplementary Fig. S3). Consistent with our previous study^30^, we showed that MUC1-induced EGFR activation and nuclear translocation.

Previous studies reported that EGFR promotes IL-6 secretion through activating downstream signaling pathways. EGFR variant III (EGFRvIII) was found to potentiate IL-1β-induced IL-6 secretion through the p38 MAPK-MK2-HuR pathway in glioblastoma cells^48^. Ligand-dependent activation of EGFR was reported to promote IL-6 expression via the NF-κB pathway in advanced-stage epithelial ovarian cancer^49^. Here, we demonstrated for the first time that EGFR directly bound to IL-6 promoter through CREB and GRβ binding sites (Fig. 3). CREB is a transcription factor widely present in the nucleus of eukaryotic cells^50^. Previous study reported that EGFR can activate ERK and p38 MAPK, which stimulate CREB-mediated IL-6 expression^51^. GRβ is an isoform of human GR, which is encoded by the *NR3C1* gene and can function as a transcription factor^52^. Silencing of GR in monocytes resulted in a significant inhibition of IL-6 production^53^. By silencing CERB and GRβ in paclitaxel-resistant cells, we verified their pivotal role in the transcriptional regulation of IL-6. Whether EGFR binds to IL-6 promoter through interaction with CREB and GRβ or through phosphorylating them needs further study. In addition, previous studies have revealed that MUC1 and EGFR can act as transcriptional coactivators and bind to multiple transcription factors such as NF-κB^49,54^. We also found that IL-6 transcription was lightly reduced by mutation of NFκB site.

Apart from both in vitro and in vivo data supporting a critical role of EGFR in MUC1-mediated paclitaxel-resistance, our clinical study showed elevated expression of MUC1, EGFR, and IL-6 in post-chemotherapy cervical cancer tissues. The association of MUC1-EGFR-IL-6 with poor disease-free survival in cervical cancer patients with chemotherapy was also found from TCGA data mining.

Collectively, our study reveals a novel mechanism, in which EGFR induces enrichment of cervical CSCs via directly upregulating IL-6 transcription in a MUC1-dependent manner (Fig. 4g), implicating erlotinib treatment as a promising approach to overcome paclitaxel-resistant CSCs in MUC1-positive cervical cancer.

## Materials and methods

### Cell culture

The human cervical cancer cell line HeLa229 and SiHa (Cell Bank, Type Culture Collection, Chinese Academy of Sciences, Shanghai, China) were maintained in RPMI1640 (Corning, NY, USA) supplemented with 10% fetal bovine serum (Gibco, Grand Island, NY, USA), 100 U/ml penicillin and 100 μg/ml streptomycin. HeLa229/TR^30^ and SiHa/TR cells were generated by exposing gradually to increasing concentrations of paclitaxel, and maintained in 25 nM and 15 nM paclitaxel separately. All cell lines were authenticated by short tandem repeat profiling and free of mycoplasma.

### Drugs and antibodies

The following drugs and antibodies were used in our experiments: paclitaxel (PTX) (Sigma-Aldrich, St. Louis, MO, USA), erlotinib (Selleck Chemicals, Houston, TX, USA), anti-MUC1 antibody (Thermo Scientific, Hudson, NH, USA), anti-phospho-EGFR Tyr1068 antibody (Cell Signaling Technology, Danvers, MA, USA), and anti-EGFR antibody (Proteintech Group, Chicago, IL, USA), anti-Histone H3 (acetyl K27) (H3K27Ac) antibody (Abcam, Cambridge, MA, USA), IL-6 antibody (Novus, Littleton, CO, USA), horseradish peroxidase (HRP)-linked secondary antibody (Cell Signaling Technology, Beverly, MA, USA), anti-β-actin antibody (Merck Millipore, Billerica, MA, USA), FITC-conjugated mouse anti-human CD227 (MUC1) (BD Pharmingen, San Diego, CA, USA), PE-conjugated mouse anti-human CD133/1 (AC133) (Mitenyi Biotec, Bergisch Gladbach, Germany), IL-6 neutralizing antibody (Sino Biological Inc., Beijing, China), and anti-human IL-8 antibody (PeproTech, Rocky Hill, NJ, USA).

### Plasmids and transfection

MUC1-deficient HeLa229/TR and SiHa/TR cells were established using a CRISPR/Cas9 system. The MUC1 gRNA sequences included 5′-GCTGCTCCTCACAG TGC-3′ targeting the first exon. The viral vectors were produced in HEK293T cells as described^30^. For MUC1 and EGFR knockdown, the scrambled shRNA, the MUC1-specific shRNA sequences, and the EGFR-specific shRNA sequences were described previously^30^. For CREB and GRβ knockdown, the scrambled shRNA, the CREB-specific shRNA sequences, and the GRβ-specific shRNA sequences were listed in Supplementary Table S1. For MUC1 overexpression, the pIRESpuro2-MUC1 and pIRESpuro2-vector have been described^30^. Transient transfections were performed according to the manufacturer’s instructions.

### Western blot

Cells were collected by trypsinization and resuspended in NETN150 lysis buffer (150 mM NaCl, 0.5% NP40, 20 mM Tris pH 8.0, 1 mM EDTA) after treated with 0 μM or 5 μM erlotinib for 48 h. Western blot was performed according to our previous study^30^.

### Sphere-formation assay

Cells were trypsinized after treated with DMSO, 5 μM erlotinib, 10 μg/ml IL-6 neutralizing antibody, 250 ng/ml IL-8 neutralizing antibody, and 5 nM paclitaxel with or without 5 μM erlotinib for 48 h. Single-cell suspensions (2000 cells/well) were plated in 24-well ultralow attachment plates (Corning, Corning, NY) in serum-free medium. DMEM/F12 serum-free medium contained 0.4% BSA (Sigma, Saint Louis, USA), supplemented with 20 ng/ml epithelial growth factor (PerpoTech, Rocky Hill, NJ), 20 ng/ml basic fibroblast growth factor (PerpoTech, Rocky Hill, NJ), 50 μg/ml insulin (Sigma, Saint Louis, USA) and 1× B27 (Gibco-Life Technologies, Carlsbad, CA, USA). Cells were incubated for 1 week, and numbers of spheres (>100 μm in diameter) were counted under a stereomicroscope (Nikon eclipse Ti, Tokyo, Japan).

### Flow cytometry assay

The expression of cell surface markers (CD227 and CD133) on cells was analyzed by flow cytometry assay. Briefly, cells were suspended in PBS containing 2% BSA. Combinations of CD227-FITC and CD133-PE or their respective isotype controls were added to the cell suspension at the concentrations recommended by the manufacturer, and then incubated at 4 °C in the dark for 30 min. The labeled cells were washed with PBS. Fluorescence was determined using a BD Biosciences LSRFortessa flow cytometer and analyzed using FlowJo software (Tree Star, Inc, CA, USA).

### Colony-forming assay

Cells were counted, plated in triplicate in six-well plates (1000 cells/well) and kept growing for 14 days. Colonies were then fixed in methanol for 15 min and stained with crystal violet staining solution for 20 min at room temperature. Then, the numbers of colonies that contained >50 cells were counted, and the results were compared. Each result was repeated three times.

### ELISA

The production of IL-1, IL-6, IL-8, IL-10, IL-12, IL-13, and IL-17 were determined using the Proteintech ELISA kits (San Diego, CA, USA). The concentrations of TNF-β1 and IFN-α in cell culture supernatants were measured using the DONGEA Elisa kit (Beijing, China) and Bio-Swamp ELISA kit (Wuhan, China) separately. ELISA was performed according to the manufacturer’s instructions. The absorption was measured at 450 nm. The protein concentration was determined by comparing the relative absorbance of the samples with the standards. Each result was repeated three times.

### Quantitative real-time PCR (RT-qPCR)

RT-qPCR was performed as described previously^30^. Primer sequences are listed in Supplementary Table S2.

### Immunofluorescence assay

Immunofluorescence assay was performed according to our recent report^30^.

### Chromatin immunoprecipitation

ChIP assay was performed as described previously^30^. Briefly, add 37% formaldehyde to cells that were cultured in 150-mm dishes; after incubation at 37 °C for 10 min, add glycine to quench unreacted formaldehyde at room temperature for 5 min. Then, harvested and sonicated cells to shear DNA, added the MUC1, EGFR, and H3K27Ac antibody separately to the supernatant fraction to cross-linked protein/DNA, elution of protein/DNA complexes, finally, reversed cross-links of protein/DNA complexes to free DNA for qPCR. Primer sequences are listed in Supplementary Table S3.

### Luciferase assay

Luciferase assay was performed as described previously^30^.

The human IL-6 promoter (−1503 to +557), IL-6 promoter (−645 to +557), and IL-6 promoter (+386 to +557) were amplified and then cloned into pGL3-basic vector (Promega, Madison, WI, USA) to construct pGL3-IL-6-promoter (−1503 to +557) luciferase reporter, pGL3-IL-6-promoter (−645 to +557) luciferase reporter and pGL3-IL-6-promoter (+386 to +557) luciferase reporter. Primer sequences are listed in Supplementary Table S4.

The pGL3-IL-6-AP-1-mt, pGL3-IL-6-NF-κB-mt, pGL3-IL-6-C/EBP-mt, pGL3-IL-6-CREB-mt, pGL3-IL-6-C/EBPβ-predicted-mt, and pGL3-IL-6-GRβ-predicted-mt (which predicted on IL-6 +386~+504 region by PROMO online. http://alggen.lsi.upc.es/cgibin/promov3/promo/promo.cgi?dirDB=TF_8.3&idCon=152986623000&getFile=factors/0.html) luciferase reporters contain point mutations on AP-1, NF-κB, C/EBP, CREB, C/EBPβ-predicted, and GRβ-predicted transcription-binding sites, respectively, by site directed PCR mutagenesis using pGL3-IL-6-Luc-wt as a template. Primer sequences are listed in Supplementary Table S5.

### Xenograft experiments

Animal protocols were in accordance with the Shanghai Medical Experimental Animal Care Guidelines. Research was approved by the Institutional Animal Care and Use Committee of Shanghai Jiao Tong University School of Medicine. Female 6- to 8-week-old athymic nude mice (BALB/c-nu/nu) were injected with indicated cells as described previously^30^. Treatment was started once the size of the xenograft reached 4 mm in diameter. Investigators were blinded to the group allocation. Tumor growth was monitored by caliper ruler every 3 days. The volume was calculated according to the formula: V = 0.52 × length × width^2^.

### Human tissue samples

Cervical tumor tissue specimens from 20 cervical cancer patients were collected during 2014 and 2017 at Renji Hospital affiliated to Shanghai Jiao Tong University School of Medicine. Signed informed consent was obtained from all the patients involved in this study, and the experimental protocols were approved by the ethical committee of Renji Hospital, according to the Declaration of Helsinki. In paired primary untreated and post-NACT, tumors were obtained to perform immunohistochemistry. All patients received paclitaxel and cisplatin treatment for one to two cycles. Detailed clinical information is summarized in Supplementary Table S6.

### Immunohistochemistry analysis

Immunohistochemistry analysis was performed as previously described^30^. The tissue images were captured on microscope (ZEISS, Jena, Germany). Two pathologists conducted the IHC scoring procedure independently, in duplicate. Score criterion of IHC was performed as follows, the percentage of staining: 0, <5%; 1, 5–25%; 2, 26–50%; 3, 51–75%; and 4, 75%–100%. The intensity of staining: 0 = negatively staining, 1 = weakly staining, 2 = moderately staining, and 3 = strongly staining. The final score was determined by multiplying the scores of the percentage of staining by the intensity of staining.

### Statistical analysis

All statistical analyses were performed by GraphPad Prism 6 (GraphPad Software, La Jolla, CA, USA). The results are shown as means ± SD and representative of three independent experiments. Differences between linked groups were evaluated by two-tailed Student's *t* test. *P < 0.05; **P < 0.01; and ***P < 0.001; ns stand for not significant.

## Supplementary information

Supplementary figure legends

Supplementary tables

Supplementary figure1

Supplementary figure2

Supplementary figure3

Supplementary figure4

Supplementary figure5

Supplementary figure6

Supplementary figure7
