# Supplementary material for: Erlotinib overcomes paclitaxel-resistant cancer stem cells by blocking the EGFR-CREB/GRβ-IL-6 axis in MUC1-positive cervical cancer
Source: Oncogenesis. 2019 Nov 26;8(12):70. doi: 10.1038/s41389-019-0179-2 (PMC6879758; doi:10.1038/s41389-019-0179-2)
Supplement: Supplementary file 1 — Supplementary figure legends [file 41389_2019_179_MOESM1_ESM.doc]

**Supplementary figure legends**

**Figure S1. Erlotinib suppresses CSCs enrichment in paclitaxel-resistant cervical cancer cells.** (A-B) Sphere formation assay (A), flow cytometry assay and colony-forming assay (B) were performed to detect the number of spheres, the percentage of CD133^+^ cells and the number of colonies in HeLa229P and HeLa229/TR cells after treated with 0 μM or 5 μM erlotinib for 48 hour (scale bar = 100 µm). (C-D) Sphere formation assay (C), flow cytometry assay and colony-forming assay (D) were performed to detect the number of spheres, the percentage of CD133^+^ cells and the number of colonies in SiHaP and SiHa/TR cells after treated with 0 μM or 5 μM erlotinib for 48 hour (scale bar = 100 µm). (E-F) Western blot (E), sphere formation assay, FCM analysis and colony-forming assay (F) were performed in HeLa229 TR/shCTL and HeLa229 TR/shEGFR cells. Data are shown of three independent experiments, mean ± SD (n = 3). P: parental; TR: paclitaxel-resistant; Erl: erlotinib.

**Figure S2. Erlotinib prevents cervical CSCs enrichment through inhibiting IL-6.** (A) Captions for collection of conditional medium form HeLa229P or HeLa229/TR cells with or without 5 μM erlotinib for 48 hour to culture HeLa229P or HeLa229/TR cells. (B) Flow cytometry assay was applied to detect the percentage of CD133^+^ cells. (C) Sphere formation assay (upper) and colony-forming assay (lower) were performed in HeLa229P and HeLa229/TR cells treated with DMSO or IL-6 neutralizing antibody (10 μg/ml) for 48 hour (scale bar = 100 µm). (D) HeLa229P and HeLa229/TR cells were exposed to the DMSO or IL-8 neutralizing antibody (250 ng/ml) for 48 hour, sphere formation assay (upper) and colony-forming assay (lower) were performed (scale bar = 100 µm). (E) Sphere formation assay was performed in SiHaP and SiHa/TR cells treated with DMSO or IL-6 neutralizing antibody (10 μg/ml) for 48 hour (scale bar = 100 µm). (F) SiHaP and SiHa/TR cells were exposed to the DMSO or IL-8 neutralizing antibody (250 ng/ml) for 48 hour, sphere formation assay was performed (scale bar = 100 µm). Data represent mean ± SD from three independent experiments (n = 3). Differences between linked groups were evaluated by two-tailed student's t-test. ***: P < 0.001; ns: not significant; P: parental; TR: paclitaxel-resistant; Erl: erlotinib; αIL-6: IL-6 neutralizing antibody; αIL-8: IL-8 neutralizing antibody.

**Figure S3. MUC1 activates EGFR to promote IL-6 expression and CSCs enrichment in paclitaxel-resistant cells.** (A-D) Western blot (A), RT-qPCR (B left), ELISA (B right), sphere formation assay (C) and colony-forming assay (D) were performed in HeLa229 TR/CTL and HeLa229 TR/CRISPR cells after treated with 0 μM or 5 μM erlotinib for 48 hour. (E) HeLa229 TR/CTL and HeLa229 TR/CRISPR cells were exposed to DMSO or IL-6 neutralizing antibody (10 μg/ml) for 48 hour, sphere formation assay was performed. (F-I) Western blot (F), RT-qPCR (G left), ELISA (G right), sphere formation assay (H) and colony-forming assay (I) were performed in SiHa TR/CTL and SiHa TR/CRISPR cells after treated with 0 μM or 5 μM erlotinib for 48 hour. Data represent mean ± SD from three independent experiments (n = 3). Differences between linked groups were evaluated by two-tailed student's t-test. ***: P < 0.001; ns: not significant; P: parental; TR: paclitaxel-resistant; Erl: erlotinib; PTX: paclitaxel.

**Figure S4. MUC1 activates EGFR to promote IL-6 expression and CSCs enrichment in parental cells upon paclitaxel treatment.** (A) HeLa229/shCTL and HeLa229/shMUC1 cells were treated with 0 nM or 5 nM paclitaxel for 48 hour. Western blot was performed to detect the expression of indicated proteins, β-actin was used as loading control. (B) HeLa229/shCTL and HeLa229/shMUC1-B cells were treated with paclitaxel (5 nM) in absence or presence of erlotinib (5 μM) for 48 hour. RT-qPCR (left) and ELISA (right) were performed. (C) Captions for collection of conditional medium form HeLa229/shCTL or HeLa229/shMUC1-B cells with or without paclitaxel treatment to culture HeLa229/shCTL or HeLa229/shMUC1-B cells. (D) Sphere formation assay (upper) and colony-forming assay (lower) were performed in HeLa229/shCTL and HeLa229/shMUC1-B cells after treated with 0 nM paclitaxel, 5 nM paclitaxel, 5 μM erlotinib or 5 nM paclitaxel combined with 5 μM erlotinib for 48 hour (scale bar = 100 µm). (E) Sphere formation assay was performed in HeLa229/shCTL and HeLa229/shMUC1-B cells after treated with 0 nM paclitaxel, 5 nM paclitaxel, 10 μg/ml αIL-6 or 5 nM paclitaxel combined with 10 μg/ml αIL-6 for 48 hour (scale bar = 100 µm). Data are shown of three independent experiments, mean ± SD (n = 3). Differences between linked groups were evaluated by two-tailed student's t-test. ***: P < 0.001; ns: stand for not significant; shM-B: shMUC1-B; PTX: paclitaxel; TR: paclitaxel-resistant; Erl: erlotinib; αIL-6: IL-6 neutralizing antibody.

**Figure S5. EGFR induces IL-6 transcription through CREB and GRβ binding sites.** (A) Immunofluorescence staining of MUC1 (green) and EGFR (red) in HeLa229P, HeLa229/TR treated with or without erlotinib. (B) Transfected with MUC1 to HeLa229/shMUC1-B cells and exposed to 0 nM or 5 nM paclitaxel for 48 hour, then immunofluorescence was performed to detect the distribution of MUC1 (green) and EGFR (red), nuclei were stained blue with DAPI. (C) The mRNA levels of CREB in HeLa229 TR/shCTL and HeLa229 TR/shCREBs cells were detected by RT-qPCR. (D) The mRNA levels of GRβ in HeLa229 TR/shCTL and HeLa229 TR/shGRβs cells were detected by RT-qPCR. P: parental; TR: paclitaxel-resistant; TR/Erl: paclitaxel-resistant/erlotinib; PTX: paclitaxel.

**Figure S6. Coadministration of EGFR inhibitor with paclitaxel prevents MUC1-expressing tumor relapse.** (A**-**D) HeLa229/shCTL cells or HeLa229/shMUC1 cell were subcutaneously injected to nude mice. Nude mice with tumors of 4 mm in diameter were randomized to 2 groups (n = 6 per group) and injected with PBS or paclitaxel (40 mg/kg) intraperitoneally every 3 days for 15 days. Tumor sizes were measured every 3 days and the tumor volumes were calculated (A-B). Immunohistochemical staining (C), RT-qPCR (D left), ELISA (D middle) and flow cytometry assay (D right) were performed (scale bar = 50 µm). (E-F) HeLa229 TR/CTL or HeLa229 TR/CRISPR cells were subcutaneously injected to nude mice. When the tumors reached 4 mm x 4 mm, the mice were randomized to groups (n = 6 per group) and injected drugs as indicated intraperitoneally every 3 days for 15 days and the tumor volume was calculated (E). At day 33, mice were euthanized and tumors were excised. Tumor weight (F upper) and body weight (F lower) were measured. Differences between linked groups were evaluated by two-tailed student's t-test. *: P < 0.05; ***: P < 0.001; ns: not significant; PTX: paclitaxel; Erl: erlotinib.

**Figure S7. Activation of MUC1-EGFR-IL-6 signaling correlates with poor disease-free survival of cervical cancer patients with chemotherapy.** (A) Representative IHC staining images of one paired pre- and post-NACT cervical cancer tumor tissues (scale bar = 50 µm). (B) IHC score of MUC1, EGFR and IL-6 of each pre- and post-NACT tissues correspondingly (n = 20). (C) The correlation between MUC1 and EGFR, MUC1 and IL-6, or EGFR and IL-6 expression were analyzed in pre-NACT cervical cancer tissues (n = 20). PTX: paclitaxel; NACT: neoadjuvant chemotherapy.
