# Supplementary material for: Erlotinib overcomes paclitaxel-resistant cancer stem cells by blocking the EGFR-CREB/GRβ-IL-6 axis in MUC1-positive cervical cancer
Source: Oncogenesis. 2019 Nov 26;8(12):70. doi: 10.1038/s41389-019-0179-2 (PMC6879758; doi:10.1038/s41389-019-0179-2)
Supplement: Supplementary file 2 — Supplementary tables [file 41389_2019_179_MOESM2_ESM.doc]

**Supplementary tables**

**Table S1. Target sequences of shRNA:**

| shCTL | 5’-CGCTTACCGATTCAGAATGG-3’ |
| --- | --- |
| shCREB-A | 5’-AGCAACCAAGTTGTTGTTC-3’ |
| shCREB-B | 5’-GTCACATTCTCATTGTGAA-3’ |
| shGRβ-A | 5’-TGACTGTAGCTGTAGGTGA-3’ |
| shGRβ-B | 5’-CCAGAAAGCACATCTCACACATTAATCTG-3’ |

**Table S2. Primer sequences for RT-qPCR:**

| IL-1 forward | 5’-ATGATGGCTTATTACAGTGGCAA-3’ |
| --- | --- |
| IL-1 reverse | 5’-GTCGGAGATTCGTAGCTGGA-3’ |
| IL-6 forward | 5’-GAGCAGGCACCCCAGTTAAT-3’ |
| IL-6 reverse | 5’-ATTTGTGGTTGGGTCAGGGG-3’ |
| IL-8 forward | 5’-TTTTGCCAAGGAGTGCTAAAGA-3’ |
| IL-8 reverse | 5’-AACCCTCTGCACCCAGTTTTC-3’ |
| IL-10 forward | 5’-GACTTTAAGGGTTACCTGGGTTG-3’ |
| IL-10 reverse | 5’-TCACATGCGCCTTGATGTCTG-3’ |
| IL-12 forward | 5’-AAAGGACATCTGCGAGGAAAGTTC-3’ |
| IL-12 reverse | 5’-CGAGGTGAGGTGCGTTTATGC-3’ |
| IL-13 forward | 5’-CCTCATGGCGCTTTTGTTGAC-3’ |
| IL-13 reverse | 5’-TCTGGTTCTGGGTGATGTTGA-3’ |
| IL-17 forward | 5’-TCCCACGAAATCCAGGATGC-3’ |
| IL-17 reverse | 5’-GGATGTTCAGGTTGACCATCAC-3’ |
| TNF-β1 forward | 5’-GGCCAGATCCTGTCCAAGC-3’ |
| TNF-β1 reverse | 5’-GTGGGTTTCCACCATTAGCAC-3’ |
| IFN-α forward | 5’-GCTTGGGATGAGACCCTCCTA-3’ |
| IFN-α reverse | 5’-CCCACCCCCTGTATCACAC-3’ |
| CREB forward | 5’-CACCAGGAGTGCCAAGGATT-3’ |
| CREB reverse | 5’-CTGCTGCATTGGTCATGGTT-3’ |
| GRβ forward | 5’-TGTGTTTTGCTCCTGATCTGA-3’ |
| GRβ reverse | 5’-TGAGATGTGCTTTCTGGTTTT-3’ |
| 18S rRNA forward | 5’-GTAACCCGTTGAACCCCATT-3’ |
| 18S rRNA reverse | 5’-CCATCCAATCGGTAGTAGCG-3’ |

**Table S3. Primer sequences for** **ChIP:**

| -4700~-4547 forward | 5’-AAGACGTCATAACCAGGCCA-3’ |
| --- | --- |
| -4700~-4547 reverse | 5’-TGAGTTCAGTGTCATCAGCAG-3’ |
| -3676~-3511 forward | 5’-ACAAACTTCTTACGACTTGGCA-3’ |
| -3676~-3511 reverse | 5’-TGTAGTGTTGTGCTCTTTCTCT-3’ |
| -2582~-2481 forward | 5’-AAGTGCCATGCTGCGATGT-3’ |
| -2582~-2481 reverse | 5’-GCCTGATGCTTCTGGGGTC-3’ |
| -656~-489 forward | 5’-AACCTCCTCTAAGTGGGCTG-3’ |
| -656~-489 reverse | 5’-TTCTTCTGTGTTCTGGCTCTCCC-3’ |
| +386~+504 forward | 5’-ATTCCAAAGATGTAGCCGCC-3’ |
| +386~+504 reverse | 5’-AAGCCTACCCACCTCCTTTC-3’ |

**Table S4. Primer sequences for IL-6 promoters：**

| -1503 to +557 forward | 5’-CGACGCGTCTAAAAATGAAACCATCCAGC-3’ |
| --- | --- |
| -1503 to +557 reverse | 5’-AAAAGCTTACACGCAGGGGCAAGGGGAAC-3’ |
| -645 to +557 forward | 5’- AGACGCGTAGTGGGCTGAAGCAGGTGAA-3’ |
| -645 to +557 reverse | 5’-AAAAGCTTACACGCAGGGGCAAGGGGAAC-3’ |
| +386 to +557 forward | 5’-CCGACGCGTATTCCAAAGATGTAGCCG-3’ |
| +386 to +557 reverse | 5’-AAAAGCTTACACGCAGGGGCAAGGGGAAC-3’ |

**Table S5. Primer sequences for site directed PCR mutagenesis:**

| pGL3-IL-6-AP-1-mt forward | 5’-GCCAAAGTGCTGAGTTGCTAATAAAAG-3’ |
| --- | --- |
| pGL3-IL-6-AP-1-mt reverse | 5’-TTATTAGCAACTCAGCACTTTGGCATGT-3’ |
| pGL3-IL-6-NF-κB-mt forward | 5’-CAAAGATTTATCAAATGTAATATTTTCCCATGAGTCTCAAT-3’ |
| pGL3-IL-6-NF-κB-mt reverse | 5’-GAAAATATTACATTTGATAAATCTTTGTTGGAGGGTGAGG-3’ |
| pGL3-IL-6-C/EBP-mt forward | 5’-GGACGTCACACTACAAACTCTTAATAAGGTT-3’ |
| pGL3-IL-6-C/EBP-mt reverse | 5’-ATTAAGAGTTTGTAGTGTGACGTCCTTTAGC-3’ |
| pGL3-IL-6-CREB-mt forward | 5’-CCATGCTAAAGGTGGTCACATTGCACAATCTT-3’ |
| pGL3-IL-6-CREB-mt reverse | 5’-CAATGTGACCACCTTTAGCATGGCAAGACAC-3’ |
| pGL3-IL-6-C/EBPβ-mt forward | 5’-GAACGAATTGAGATACAGATTCGGTACATCCTC-3’ |
| pGL3-IL-6-C/EBPβ-mt reverse | 5’-GTACCGAATCTGTATCTCAATTCGTTCTGAAG-3’ |
| pGL3-IL-6-GRβ-mt forward | 5’-GAATTGACAAACGACTACGGTACATCCTCGACG-3’ |
| pGL3-IL-6-GRβ-mt reverse | 5’-GATGTACCGTAGTCGTTTGTCAATTCGTTCTG-3’ |

**Table S6. Clinical information of cervical cancer patients.**

| Patients Characteristics (n = 20) | | | |
| --- | --- | --- | --- |
| Median age | 51.5（39-76） | | |
| Pathological type | Squamous cell carcinoma | | 95% |
|  | Adenocarcinoma | | 5% |
| FIGO stage | IB2 | | 15% |
|  | IIA1 | | 20% |
|  | IIA2 | | 50% |
|  | IIB | | 15% |
| Lymph node metastasis | Yes | | 25% |
|  | No | | 75% |
| NACT cycle | 1 | | 30% |
|  | 2 | | 70% |
| IHC MUC1 | Before | | After |
|  | - | 10% | 10% |
|  | + | 90% | 55% |
|  | ++ | 0 | 35% |
| IHC EGFR | Before | | After |
|  | - | 45% | 10% |
|  | + | 55% | 90% |
| IHC IL-6 | Before | | After |
|  | - | 15% | 20% |
|  | + | 85% | 70% |
|  | ++ | 0 | 10% |

The clinical information including age, pathological type, FIGO stage, lymph node metastasis and IHC of MUC1, EGFR, IL-6 were shown. The IHC final score was determined by multiplying the scores of the percentage of staining with the intensity of staining. -: 0; +: 1~4; ++: 5~8; FIGO: International Federation of Gynecology and Obstetrics.
