# Supplementary figures and images for: Erlotinib overcomes paclitaxel-resistant cancer stem cells by blocking the EGFR-CREB/GRβ-IL-6 axis in MUC1-positive cervical cancer
Source: Oncogenesis. 2019 Nov 26;8(12):70. doi: 10.1038/s41389-019-0179-2 (PMC6879758; doi:10.1038/s41389-019-0179-2)

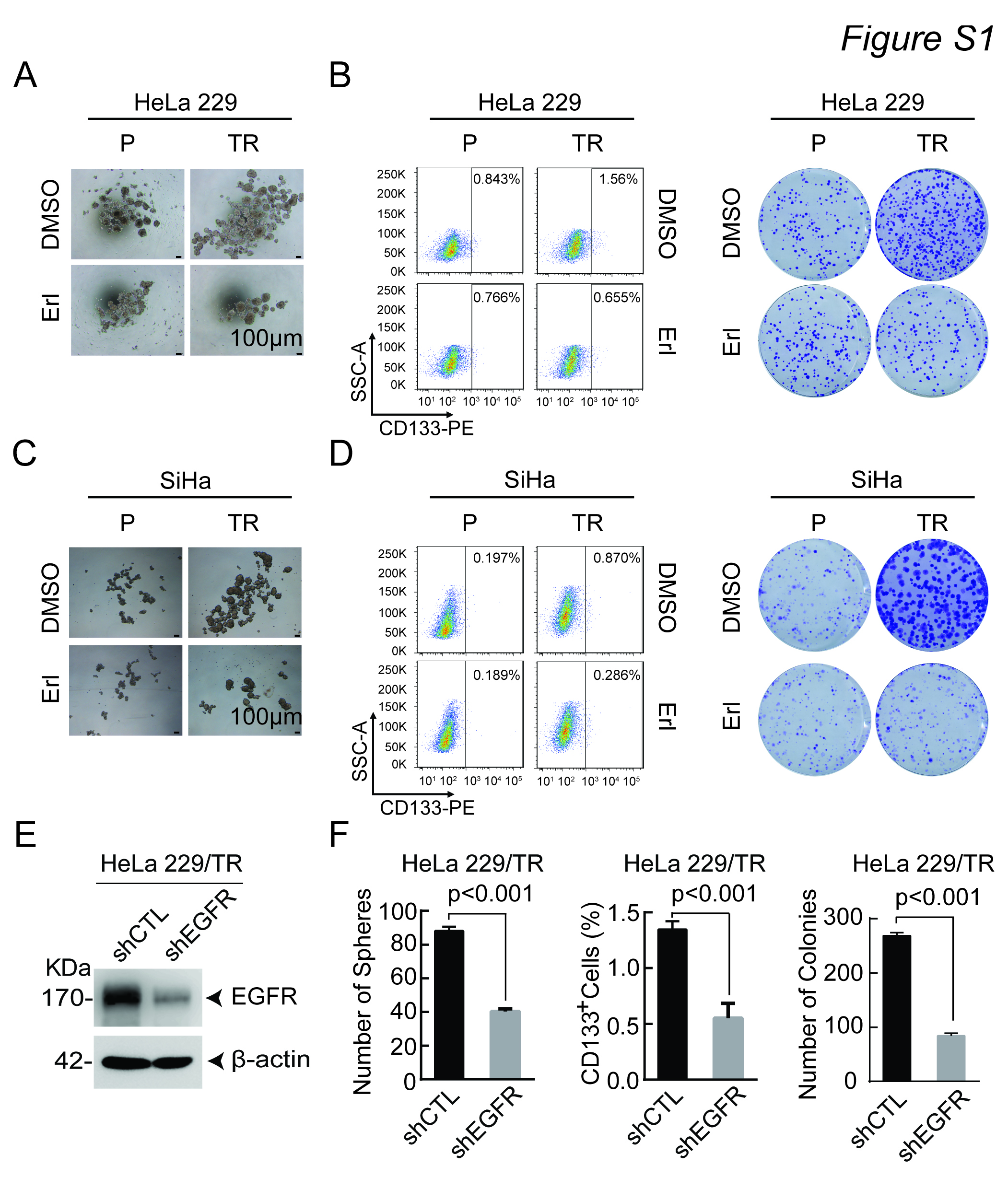

Supplement: Supplementary file 3 — Supplementary figure1 [file 41389_2019_179_MOESM3_ESM.jpg]

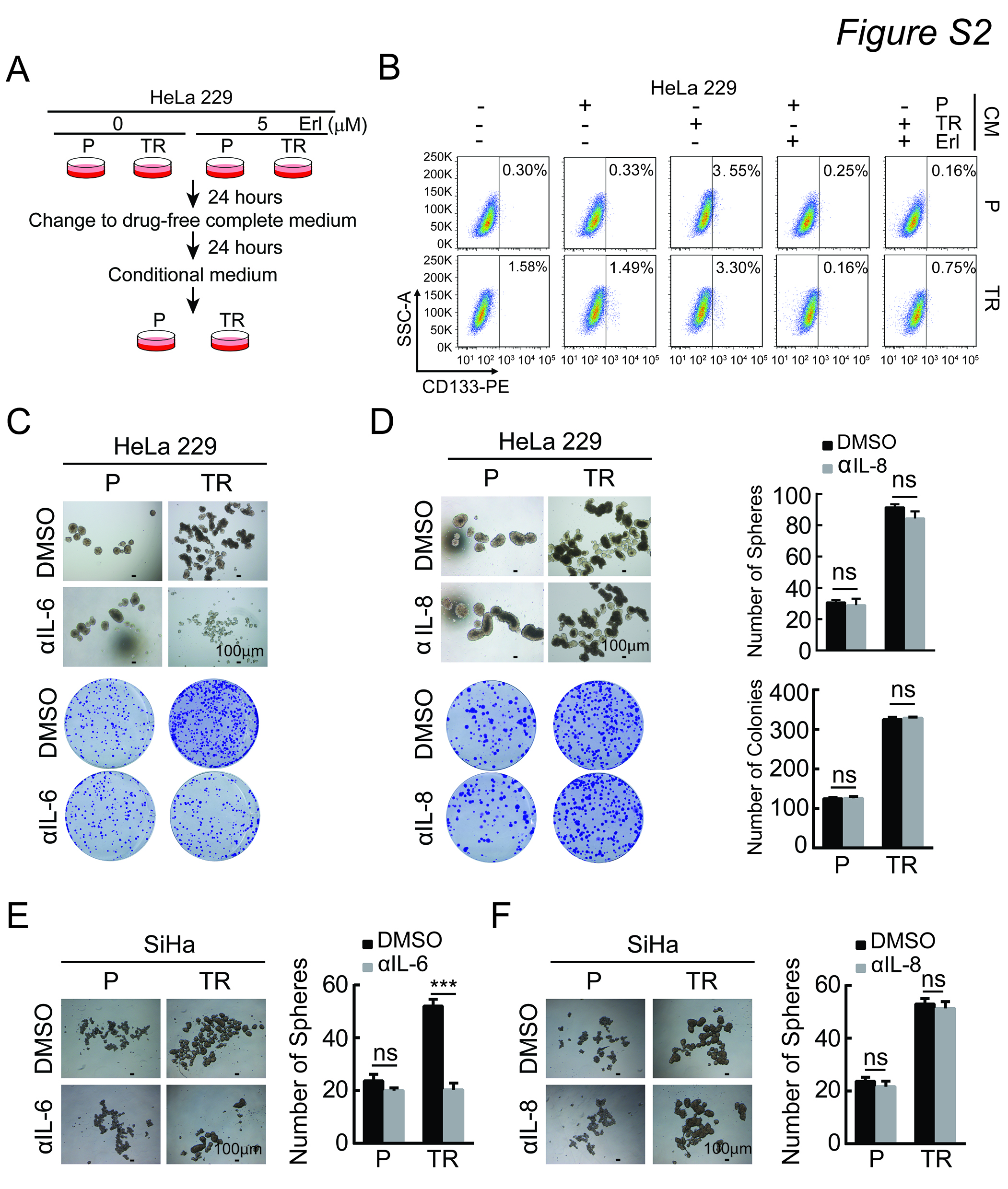

Supplement: Supplementary file 4 — Supplementary figure2 [file 41389_2019_179_MOESM4_ESM.jpg]

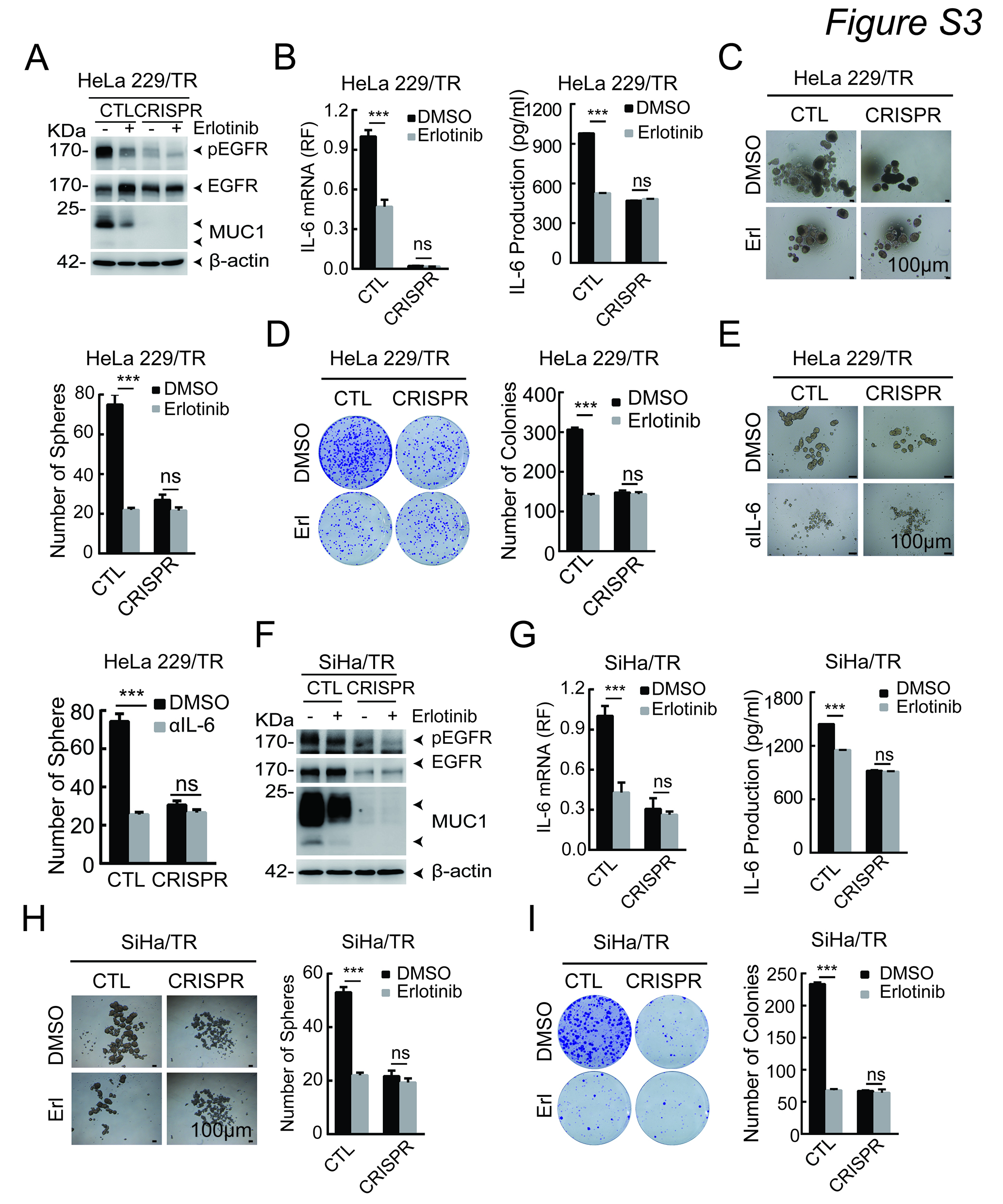

Supplement: Supplementary file 5 — Supplementary figure3 [file 41389_2019_179_MOESM5_ESM.jpg]

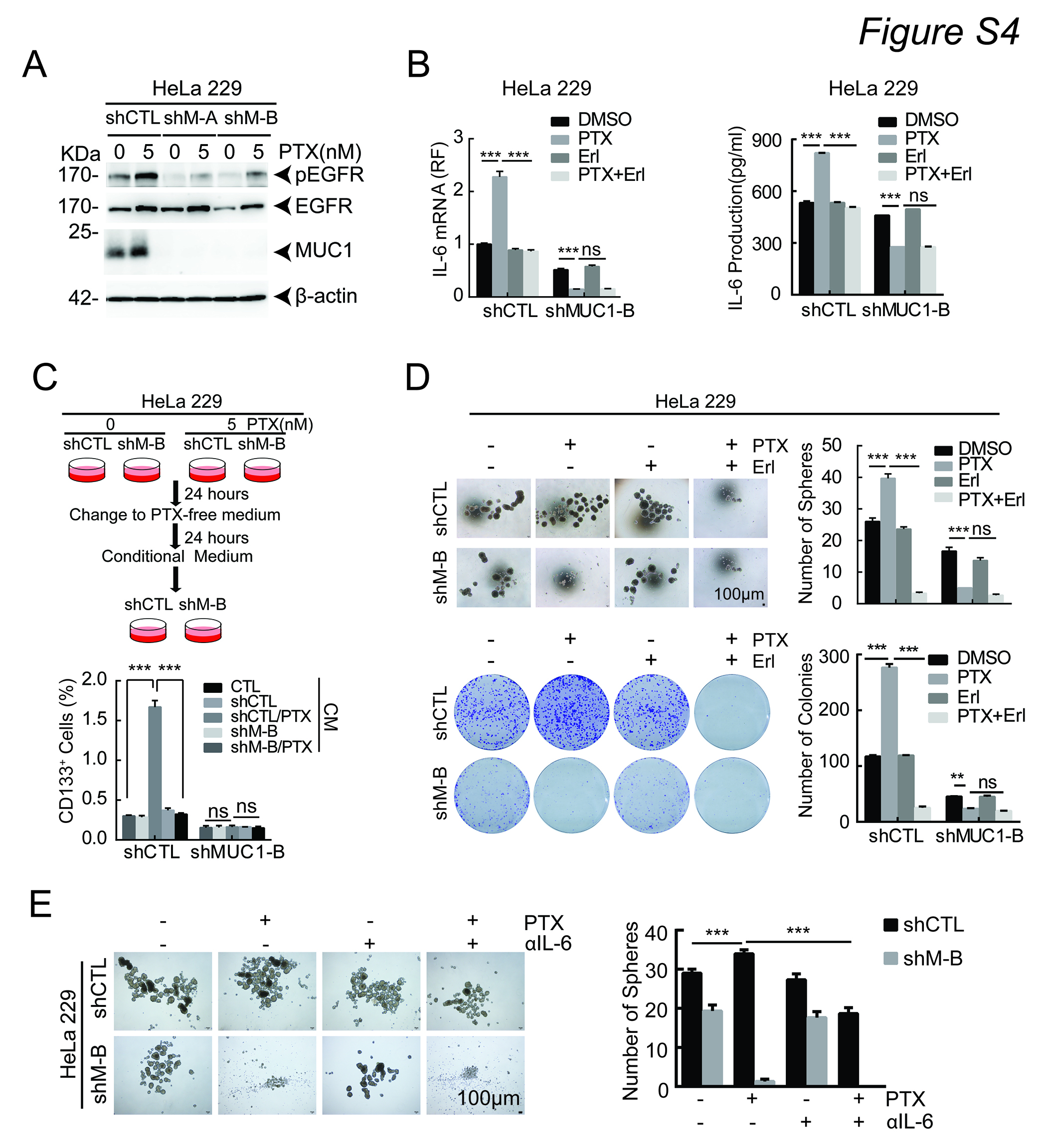

Supplement: Supplementary file 6 — Supplementary figure4 [file 41389_2019_179_MOESM6_ESM.jpg]

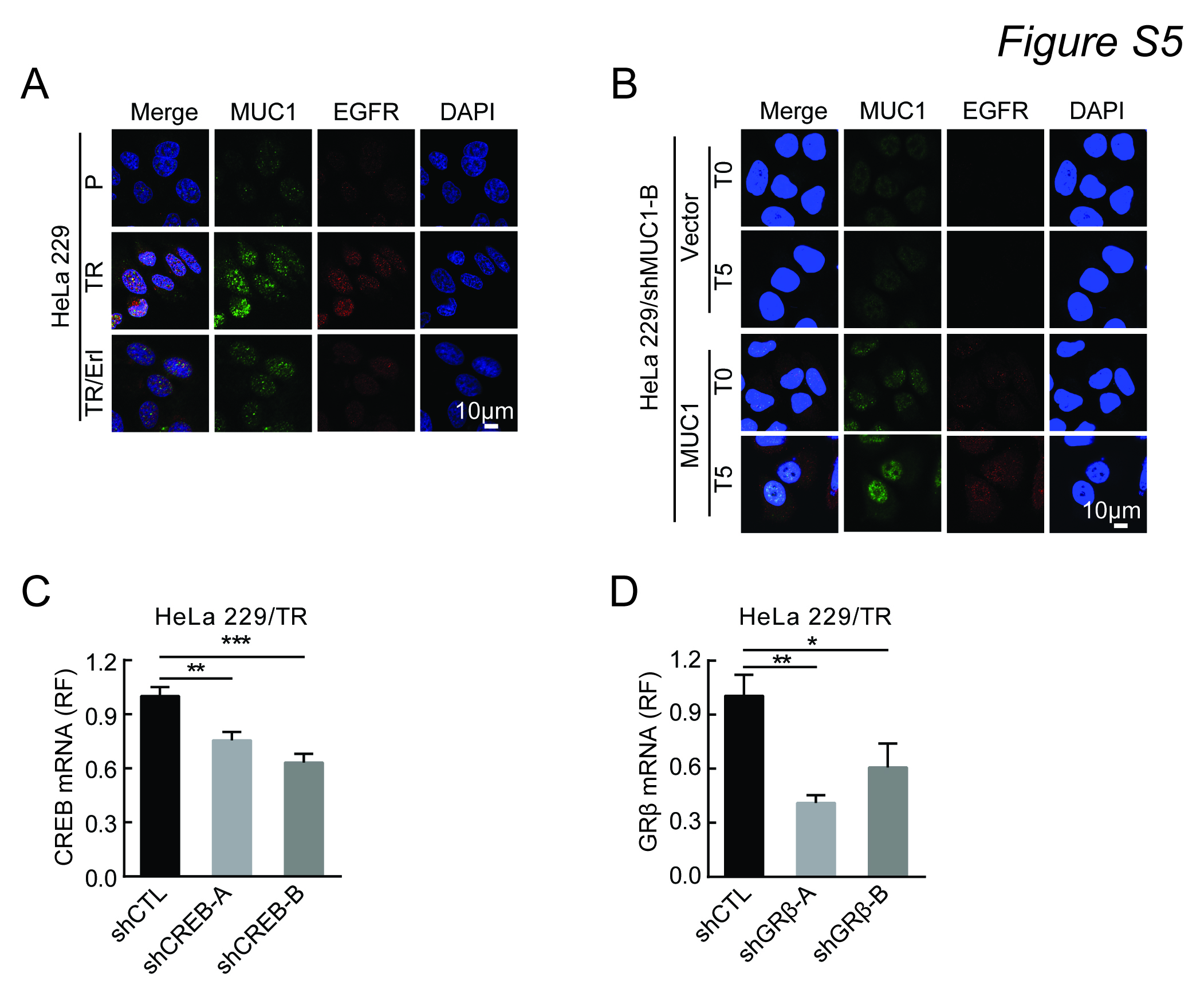

Supplement: Supplementary file 7 — Supplementary figure5 [file 41389_2019_179_MOESM7_ESM.jpg]

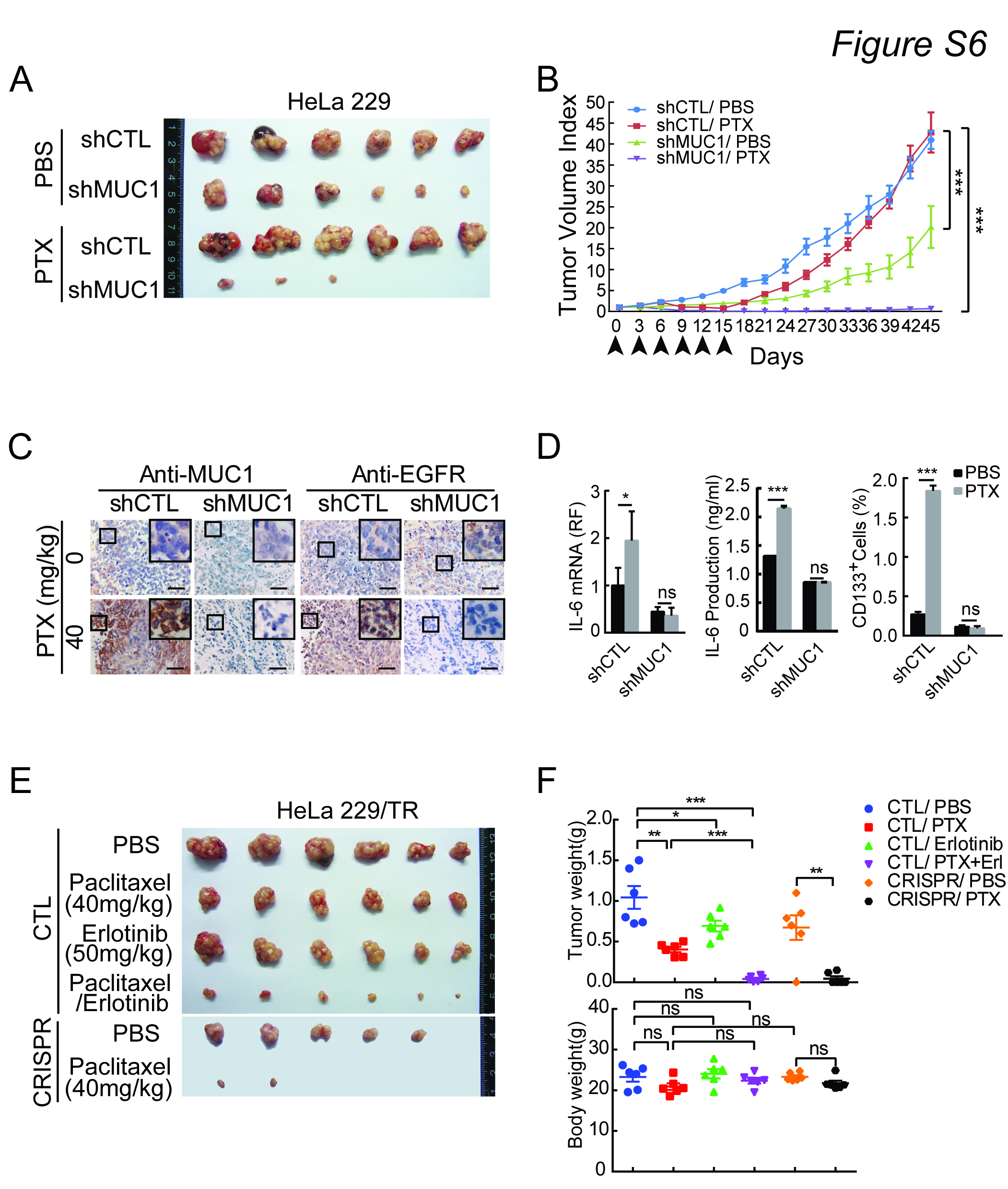

Supplement: Supplementary file 8 — Supplementary figure6 [file 41389_2019_179_MOESM8_ESM.jpg]

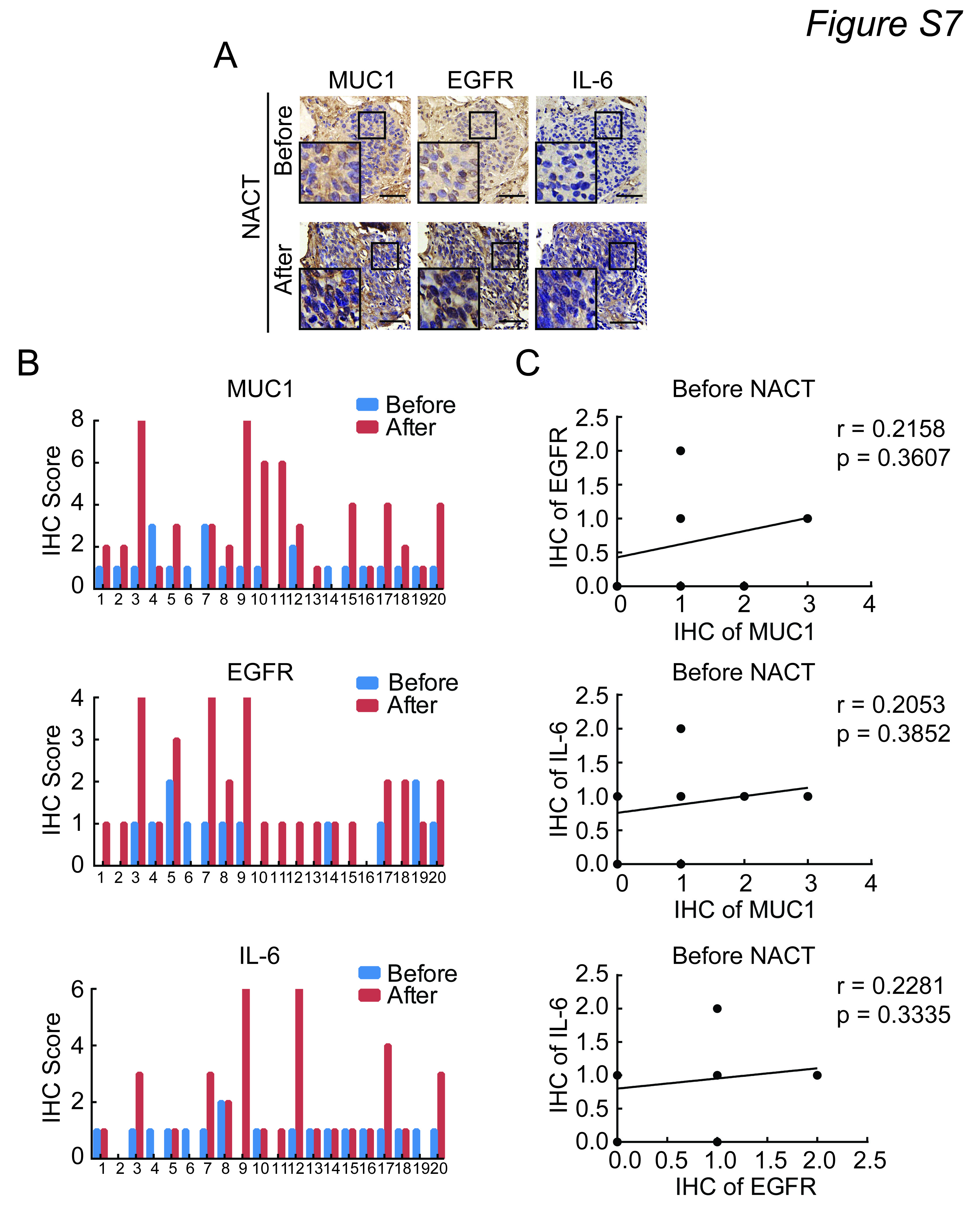

Supplement: Supplementary file 9 — Supplementary figure7 [file 41389_2019_179_MOESM9_ESM.jpg]
